# Central odontogenic fibroma: a case report with long-term follow-up

**DOI:** 10.1186/1746-160X-6-20

**Published:** 2010-08-13

**Authors:** Marco T Brazão-Silva, Alexandre V Fernandes, Antônio F Durighetto-Júnior, Sérgio V Cardoso, Adriano M Loyola

**Affiliations:** 1 General Pathology master degree program, Federal University of Triângulo Mineiro, Uberaba, MG, Brazil; 2 Oral Diagnosis Section, School of Dentistry, Federal University of Uberlândia, Uberlândia, MG, Brazil; 3 Oral Pathology Section, School of Dentistry, Federal University of Uberlândia, Uberlândia, MG, Brazil

## Abstract

An osteolytic tumour of the mandible with prominent expansive growth on the alveolar ridge and displacement of the involved teeth is described in a 28-year-old man. The lesion was diagnosed as a central odontogenic fibroma, an uncommon benign neoplasm derived from dental apparatus, and was removed by curettage. The patient remains asymptomatic after thirteen years of follow-up, which supports the claimed indolent behavior of this poorly documented disease and the adequacy of a conservative surgical treatment.

## Introduction

Central odontogenic fibroma (COF) is an uncommon benign neoplasm composed by varying amounts of inactive-looking odontogenic epithelium embedded in a neoplastic mature and fibrous stroma [1-12]. The lesion may evolve from a dental germ (dental papilla or follicle) or from the periodontal membrane, and therefore is invariably be related to the coronal or radicular portion of teeth [2,3]. Due to its non-exclusive histological features, this lesion may be confused with other entities, such as hyperplastic dental follicles, odontogenic myxomas, and desmoplastic fibromas, which highlight the importance of clinicopathological correlation in the diagnosis of odontogenic fibromas [2,3,7,12,13]. Finally, there is little information regarding long term results after surgical treatment of this lesion. We describe a COF in the right canine/premolar area of the mandible in an adult male. In addition, we discuss relevant issues about the origin, diagnosis and management of the present lesion.

## Case report

**Figure 1:**
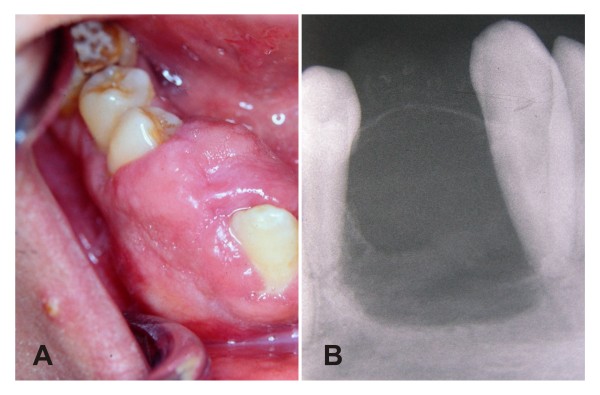
**A - Intra-oral view demonstrating gingival swelling in the alveolar ridge between canine and premolar teeth**. B - Periapical radiography demonstrating a radiolucent osteolitic lesion with internal osseous septa, and points of calcifications on buccal surface. Besides, shows a thin radiopaque line around the superior aspect of the lesion and resorption of the lamina dura without radicular resorption.

A 28-year-old man presented with a painless periodontal swelling in the right side of the mandible. The patient reported five years of evolution, with moderate discomfort during mastication as the only relevant symptom. Oral examination revealed a 2.5 cm sessile tumour on the right side of the alveolar ridge of the mandible, between canine and first premolar (Figure 1.A). These teeth were displaced by the lesion without relevant mobility and positive responses were obtained to thermal test of pulp vitality. The lesion had a firm consistency and was covered by a normal overlying mucosa. There were no clinical signs of inflammation in spite of that surface indentations caused by their upper right canine. On radiographs, it was evidenced a rounded unilocular radiolucent alteration surrounded by a thin radiopaque membrane, with some discrete radiopaque spots. There was not radicular resorption albeit the lamina dura of the affected teeth was not apparent in the proximal aspect to the lesion (Figure 1.B). Puncture of the tumor did not revealed liquid content, and a punch biopsy was performed to obtain a fragment with a myxoid appearance.

**Figure 2:**
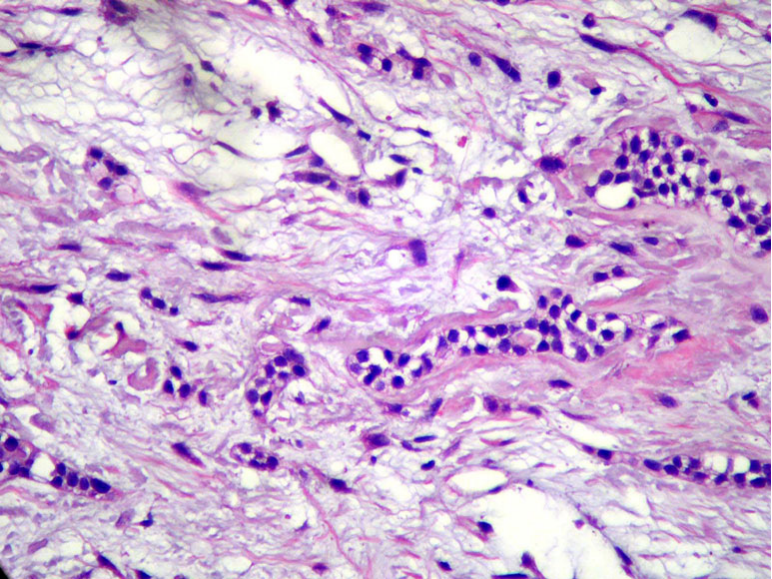
**Microscopic view demonstrating a lesion constituted by fibrous connective tissue and abundant nests and strands of inactive-looking OE usually surrounded by basophilic extra-cellular substance (1000×, hematoxilin-eosin)**.

Microscopically, the sample consisted in a fibrous connective tissue alternated with more vacuolated myxomatous areas. Individual nuclear morphology of the fibroblasts varied from spindle shaped to stellate. Abundant nests and strands of odontogenic epithelium were found, often with a clear or vacuolated cytoplasm, sometimes surrounded by juxtaepithelial hyalinization (Figure 2). Calcification, inflammatory cells and mitotic activity were not observed. Correlation of clinical, radiographic and histopathological features leaded to the diagnosis of central odontogenic fibroma.

The lesion was then entirely removed by curettage under local anesthesia. The microscopic evaluation of this material reveals the same features of the previous biopsy. All options for rehabilitation were given to the patient, who choice to use a removable partial denture. There was not any relevant event after thirteen years of follow-up.

## Discussion

Up to the present, specific information from approximately 80 patients were reported as single cases or case series in English literature [5,6,8-12,14-17]. It described a female predilection of 2.8:1, usually ranging from patients among the second and sixth decade of life. Lesions are similarly found in maxilla and mandible, most of them in the anterior region of the jaws. Tumors are asymptomatic, and claims which exist, are related to their slow growing behavior that displaces and might cause mobility of the adjacent teeth [5-21]. Our patient only claimed the lesion after 5 years, complaining only the discomfort caused by that mass, without mobility or pain.

There are two types of COF. The simple type of COF is composed by a delicate fibrous connective tissue with considerable ground substance yielding a fibromyxoid quality. Seldom rests of small and round odontogenic epithelium, often with vacuolated cytoplasm, may or may not be found [3]. In this sense it might be identical to a hyperplastic dental of an impacted tooth. Since an enlarged but narrow and well-circumscribed radiolucent area should be better regarded as a hyperplastic dental follicle, a lesion depicting persistent, progressive growth, sometimes with calcifications, is compatible with a tumor [2,3,7]. Our case was compatible with the so-called odontogenic fibroma complex type (or WHO type). This variant shows abundant islands and strands of apparently inactive odontogenic epithelium and spindle or stellate fibroblasts. Their parenchyma is composed by a connective tissue constituted by interposed bundles of collagen alternating with less cellular and less fibrous regions [1-3]. Irregular calcifications resembling dysplastic cementum, osteoid or dysplastic dentin is also present at variable amounts.

All fibrous lesions of the jaws should be considered to make a safe diagnosis, attempting to both clinical and histopathological aspects, as summarized in table 1. Various papers reported the importance to include the Desmoplastic fibromas (DF) as diagnostic hypothesis against fibrous lesions of the jaw bones [2,3,12,13,20]. DF is locally aggressive and invasive, often treated with limb spearing resections. Histologically, DFs are usually far less cellular, with more spindle shaped cells and with intensely collagenous stroma [12]. Besides, the findings such as younger age, ill-defined margins, cortical perforation, pathologic fracture and fast growth would be useful to diagnose DF instead of COF [7,12,20,21].

**Table 1:** Differential diagnosis: Odondogenic fibroma and similar fibrous lesions of jaws.

Features	Central odontogenic fibroma	Desmoplastic fibroma	Odontogenic myxoma	Ameloblastic fibroma	Adenomatoid odontogenic tumor
*Origin*	odontogenic ectomesenchyme	Fibroblastic/myofibroblastic	odontogenic ectomesenchyme	Odontogenic epithelium and odontogenic ectomesenchyme	Odontogenic epithelium
*Pathology*	Interwoven bundles of collagen embedding variable amount of scattered fibroblasts. Many nests and strands of inactive-looking OE** and calcifications can be found [1-3].	Interlaced bundles and whorled aggregates of densely collagenous tissue containing uniform spindled and elongated fibroblasts/myofibroblasts [2].	Stellate and spindle-shaped cells in a rich myxoid or mucoid stroma with few collagen fibrils. Few OE islands may be present [3,27].	Branching and anastomosing proliferative OE with peripheral rim of columnar cells in a primitive connective tissue stroma without hard tooth formations [23].	Variably sized solid nodules of cuboidal OE conspicuously with duct-like structures. Eosinophilic amorphous material called "tumor droplets" can be found [28,29].
*Presentation*^#^	1.5% of odontogenic tumors [4] Age: 34.9+19.6 [12] M:F *= 1:2.8 [12] Maxilla and mandible in equal proportions, being most affected posterior (73.5%) and anterior (73.5%) regions, respectively [5,11].	Less than 1% of bone tumors [21,30] Age: 15.1+12 [12,30] M:F = 1:1.2 [7,12] 15% may be painfull [21] Locally invasive and aggressive Almost mandible (84%), and in posterior portion of both jaws [21,30]	3-20% of odontogenic tumors [4] Age: 31.3 [31] M:F = 1:2.3 [27] 25% may be painful [27] Locally invasive and aggressive Mandible (63%) at posterior region and maxilla at premolar region [31].	1.6% of odontogenic tumors [3] Age: 9.6 [23] M:F = 1.26:1 Expansive growth Mandible (80.5%) posterior (73.5%) [23].	1.7-7% of odontogenic tumors^9, OMS^ Age:13.2 [28] M:F = 1:2.6 [28] The absence of a tooth is observed Maxilla (twice mandible) at anterior region (92.3%) [32]
*Radiology*	Well-defined radiolucency, unilocular in smaller (average of 2.2cm) and multilocular in larger (average of 4.2cm). Pinpoint calcifications may be present in 12% [3].	Well-defined, almost multilocular radiolucency (76%), more likely to involve bone expansion and boundary destruction [21].	Multilocular (60-80%) as "honeycomb", "soap bubble" or "tennis racket" aspect with well-defined borders. Lesions lower than 4.0 cm may be unilocular [22,27,31].	Well-defined, uni/multilocular radiolucency, in most cases exhibiting a radiopaque boundary [23,24].	unilocular radiolucency associated with the crown and often part of the root of an unerupted tooth, with displacement of neighbouring teeth [28]
*Therapy/prognosis*	Curettage/excellent	Surgical resection/tendency of recurrence [21]	Surgical resection/tendency of recurrence [22]	Surgical resection/tendency of recurrence; malignant transformation in 11.4% [23,25].	Curettage/excellent [28,29,32]

^# ^All tumors generally depicted asymptomatic swellings. *M:F = Masculine:Feminine.**Odontogenic epithelium.

Similarities between odontogenic myxomas and COF simple type leaded to the disputed hypothesis that the latter entity would merely represent the most collagenous variant of the histological spectrum of the odontogenic myxomas, the so-called myxofibroma [1]. The clinical history of rapid growth with expansive and invasive behavior, associated with the surgical aspect of a sticky or gelatinous tissue, is compatible with myxomas/myxofibromas. Radiographic aspects might be similar, but the aspect of ameloblastoma (soap bubbles) was expected in larger myxomas [22]. Histologically, the abundance of collagen and greater celullarity favors the diagnosis of COF, but an association with clinic-radiographic aspects may be done to exclude the hypothesis of the variant myxofibroma [3].

Ameloblastic fibromas are distinguished from COF by the fact that both the epithelial and mesenchymal components are neoplastic, while in COF, is only the mesenchymal [3,23]. It usually affect the canine to molar region, the tumor grows slowly and painlessly, expanding the jaw, similar to the COF presentation. However histologically, the epithelial component is made up of thin branching cords or small nests of odontogenic epithelium with little cytoplasm and basophilic nuclei, often with cubical shape. Larger nests may show a central area of stellate reticulum and there are no hard tooth formations [23,24].

Recurrences are not uncommon to DF (17-72%), myxoma/myxofibroma (10-33%), and AF (33%) [21-23,25]. Thus, aggressive surgical approach must be requested for those [13,22,23]. The present case represents the largest (thirteen years) postsurgical follow up of COF. There was no relapse, substantiating that conservative surgical procedures is adequate treatments for COF. We found only four cases among the papers that with follow-up greater than nine years, with one relapse [2,26]. In spite of central odontogenic fibroma be usually easily removed, not showing any adherence to bone and/or tooth structure, the recurrences were related to insufficient curettage. Herein, because of their benign slow growth characteristic, a clinical identification of recurrence or residual disease could be identified only several years after [26]. Cryotherapy has been used in the maxillofacial region to remove neoplasias such as recurrent myxomas. This therapeutic approach is conservative and has been giving low recurrences ratio [22]. However we proved here that this is not necessary if a diagnosis of COF is well conducted.

## Conclusion

In conclusion, it is essential that oral and maxillofacial surgeons, radiologists and pathologists integrate all relevant and available information to come up with a correct diagnosis and appropriate disease management. We demonstrated that conservative surgery can be performed to treat COF, which consists in a thorough curettage of the lesion. Cytogenetic and biomolecular studies are necessary to explain the true nature and pathogenesis of these diverse similar fibrous lesions, which have so distinct behaviors.

## Consent

Written informed consent was obtained from the patient for publication of this case report and accompanying images. A copy of the written consent is available for review by the Editor-in-Chief of this journal.

## Competing interests

The authors declare that they have no competing interests

## Authors' contributions

All authors read and approved the final manuscript. MT has been involved in drafting the manuscript revising it critically for important intellectual content, and to collect the results from follow-up examinations. SV conceived of the study, and participated in its design and coordination and helped to draft the manuscript. AV and AF have made the final treatment and the clinical follow-up of case, besides substantial contributions to conception and design. AM has given the final diagnosis of case after analysis and interpretation of data.
